# Supplementary material for: Selective Blocking of Graphene Defects Using Polyvinyl Alcohol through Hydrophilicity Difference
Source: Materials (Basel). 2023 Feb 28;16(5):2001. doi: 10.3390/ma16052001 (PMC10004167; doi:10.3390/ma16052001)
Supplement: Supplementary file 1 [file materials-16-02001-s001.zip › materials-2196095-supplementary.pdf]

materials

Supporting information

Selective Blocking of Graphene Defects Using Polyvinyl Alcohol through Hydrophilicity Difference

Yoonjeong Kim [1], Yanghui Kim [1,2] and Seokhoon Ahn [1,*]

[1] Institute of Advanced Composite Materials, Korea Institute of Science and Technology (KIST), Jeonbuk 55324, Republic of Korea
[2] School of Semiconductor and Chemical Engineering, Jeonbuk National University, Jeonbuk 54896, Republic of Korea
* Correspondence: ahn75@kist.re.kr; Tel.: +82-63-219-8157

Keywords: graphene; defect healing; polyvinyl alcohol (PVA); hydrophilic interactions

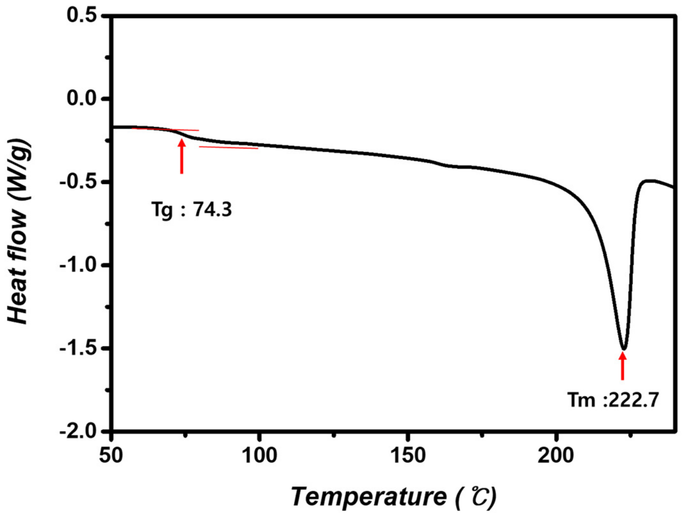

Figure S1. DSC curve of PVA powder. Glass-transition temperature (Tg) is 74.3°C, and melting temperature (Tm) is 222.7°C.
